# Wrist and finger motor representations embedded in the cerebral and cerebellar resting-state activation

**DOI:** 10.1007/s00429-021-02330-8

**Published:** 2021-07-08

**Authors:** Toshiki Kusano, Hiroki Kurashige, Isao Nambu, Yoshiya Moriguchi, Takashi Hanakawa, Yasuhiro Wada, Rieko Osu

**Affiliations:** 1 Nagaoka University of Technology, 1603-1 Kamitomioka, Nagaoka, Niigata 940-2188 Japan; 2 Research and Information Center, Tokai University, 2-3-23 Takanawa, Minato-ku, Tokyo, 108-8619 Japan; 3 Integrative Brain Imaging Center, National Center of Neurology and Psychiatry, 4-1-1 Ogawa-Higashi, Kodaira, Tokyo 187-8551 Japan; 4 Department of Integrated Neuroanatomy and Neuroimaging, Kyoto University Graduate School of Medicine, Yoshida Konoe-cho, Sakyo-ku, Kyoto, 606-8501 Japan; 5 The Advanced Telecommunications Research Institute International, 2-2-2 Hikaridai Seika, Soraku, Kyoto 619-0288 Japan; 6 Faculty of Human Sciences, Waseda University, 2-579-15 Mikajima, Tokorozawa, Saitama 359-1192 Japan

**Keywords:** fMRI, Somatotopy, Sensorimotor cortex, Cerebellum, Resting state, Cross-decoding

## Abstract

Several functional magnetic resonance imaging (fMRI) studies have demonstrated that resting-state brain activity consists of multiple components, each corresponding to the spatial pattern of brain activity induced by performing a task. Especially in a movement task, such components have been shown to correspond to the brain activity pattern of the relevant anatomical region, meaning that the voxels of pattern that are cooperatively activated while using a body part (e.g., foot, hand, and tongue) also behave cooperatively in the resting state. However, it is unclear whether the components involved in resting-state brain activity correspond to those induced by the movement of discrete body parts. To address this issue, in the present study, we focused on wrist and finger movements in the hand, and a cross-decoding technique trained to discriminate between the multi-voxel patterns induced by wrist and finger movement was applied to the resting-state fMRI. We found that the multi-voxel pattern in resting-state brain activity corresponds to either wrist or finger movements in the motor-related areas of each hemisphere of the cerebrum and cerebellum. These results suggest that resting-state brain activity in the motor-related areas consists of the components corresponding to the elementary movements of individual body parts. Therefore, the resting-state brain activity possibly has a finer structure than considered previously.

## Introduction

The brain is active even when unengaged in motor or cognitive tasks. The brain activity that occurs in the absence of any task engagement is referred to as spontaneous or resting-state brain activity (Biswal et al. 1995; Raichle 2010). Spontaneous fluctuations in brain activity, as measured by functional magnetic resonance imaging (fMRI), have physiologically significant features but not a noise component (Biswal et al. 1996; Greicius et al. 2004; Sheline and Raichle 2013; Kenet et al. 2003; Wang 2013; Albert et al. 2009). For example, several studies have reported a part of the resting-state brain activity to be associated with processes such as memory, brain function maintenance, and creative thinking, as well as indicated its usefulness as a biomarker of brain diseases (Greicius, et al. 2004; Sheline and Raichle 2013). Importantly, spontaneous fluctuations in resting-state brain activity consist of multiple components that resemble several neural substrates of more complex cognitive tasks, including anatomical parcellation such as retinotopic maps and somatotopic arrangement. This has been corroborated in studies that utilize complementary methods. For example, a study of the resting-state brain activity of anesthetized cats revealed that spontaneous fluctuations in ongoing visual cortex activity corresponded closely to orientation maps created using a voltage-sensitive dye method (retinotopic maps) (Kenet et al. 2003), and population-level patterns in the spontaneous activity of rat auditory and somatosensory cortices have previously been shown to align with the patterns that respond to direct sensory stimulation (Luczak et al. 2009). Furthermore, in the somatosensory cortices of non-human primates, a close relationship between anatomical connections from electrophysiological recordings and resting-state functional connectivity from fMRI has been established (somatotopic arrangement) (Wang et al. 2013). Collectively, these prior reports suggest that spontaneous or resting-state neural activity occurs in a replicable, reliable, and externally valid manner.

In addition, studies done in humans have revealed that resting-state brain activity often echoes activity-dependent neural activation (Biswal et al. 1995; Smith et al. 2009; Long et al. 2014). In large-scale brain networks, components estimated from resting-state brain activity by independent component analysis (ICA) are very similar to the brain activity underlying several different types of tasks (e.g., visual, auditory, and motor) (Smith et al. 2009; Laird 2011). Previous studies have also revealed that more fine-grade representations or intra-regional connectivity may occur in the visual (Wilf et al. 2017; Lu et al. 2017) and somatosensory (Long, et al. 2014) domains. In the sensorimotor-related regions, it has been previously established that task-induced brain activity in the pre- and postcentral gyri and the cerebellum are organized in close approximation to the body parts that they represent (i.e., somatotopic arrangement) (Penfield and Rasmussen 1952; Meier et al. 2008; Buccino 2001; Schieber 2001). Long et al. (2014) suggested the primary sensorimotor cortex activity in the resting state is somatotopically related to the use of body parts (e.g., the foot, hand, and tongue), and more fine-grained activity may also exist, as reported in anatomical and task-induced activation studies (Penfield and Rasmussen 1952; Meier, et al. 2008; Buccino, et al. 2001; Schieber 2001). Therefore, the specificity or fineness of these similarities between task-induced and resting-state brain activity remains unclear. Given this background, we chose to focus to wrist and finger movements, which are neighboring body parts and have widely overlapping activation areas in the brain; therefore, they are likely to have abutting cortical representations (somatotopic arrangement.)

In the present study, we aimed to confirm whether resting-state brain activity of fMRI has components similar to task-induced brain activity, even related to more discrete body parts. To achieve this goal, we used cross-decoding (Kriegeskorte 2011) to extract the task-similar resting-state brain activity in motor-related areas (primary somatosensory cortex, primary motor cortex, pre-motor area, and cerebellum) [see below and Supplementary Information (SI) for more detail]. The cross-decoding is one of multi-voxel (-variate) pattern analysis (MVPA) based on machine learning, and the sensitivity of MVPA is higher than uni-voxel (variate) statistical analyses, because it synthesizes information across multiple voxels using machine learning algorithm (Davis et al. 2014). Here, cross-decoding is a procedure of applying a decoder that has learned to classify each task based on other data, such as a combination of the movement task and the resting-state brain activity (i.e., extracting the similarity of test data to the training data). The cross-decoding outputs the similarity between the training data (movement task) and the test data (resting state).

The cross-decoding consists of two steps. The first step is extracting the characteristic multi-voxel patterns corresponding to the specific brain activity induced by each task. Several studies have shown that machine learning can estimate correspondence between several tasks (task labels) and task-induced brain activities (data) (Haynes and Rees 2005a, b; Haynes and Rees 2005a, b; Kamitani and Tong 2005, 2006; Norman, et al. 2006; Ogawa and Imamizu 2013). In fact, several studies have also adopted this approach to identify specific information represented in the resting-state brain activity (Deuker 2013; Kurashige et al. 2018; Schapiro et al. 2018).

The second step is measuring the similarity between the characteristic multi-voxel patterns of the first step and the task-similar resting-state brain activity. We defined the output of cross-decoding as a task-relevancy index (RI.) As characteristic of the decoder, RI is large when input data are similar to the training data, whereas RI is small (close to the decision boundary: ~ 0) when input data are not similar to training data. Thus, we evaluated how far the RI is from the task-dissimilar brain activity (the decision boundary) and examined the existence of the task-similar activity of wrist and finger movements in the resting-state brain activity.

## Materials and methods

### Subjects

Twenty-one healthy right-handed adult men (20–33 years; mean 23.4 years) participated in the present study. They had no history of psychiatric or neural disease and provided informed consent documents before the experiment. The Institutional Ethics Committee of the Advanced Telecommunications Research Institute International and the National Center of Neurology and Psychiatry (NCNP), in accordance with the tenets of the Declaration of Helsinki, approved of this study.

### MRI data acquisition

All structural and functional images were acquired using a Siemens Verio 3-Tesla MR scanner (Siemens, Erlangen, Germany). Here, the structural image was a three-dimensional T1-weighted image (magnetization-prepared rapid gradient-echo imaging: MPRAGE) with the following parameters: 192 slices; matrix size, 256 × 256; TR, 1900 ms; TI, 900 ms; TE, 2.52 ms; flip angle 9 degrees; voxel size, 0.97 × 0.97 × 0.97 mm. Functional images were scanned via echo-planar imaging (EPI) with the following parameters: 39 slices; matrix size, 64 × 64; TR, 3000 ms; TE, 40 ms; flip angle 90°; voxel size, 3 × 3 × 3 mm; slice gap 0.6 mm. All subject instructions were projected from outside the scanner room onto a mirror located at the scanner bore. Note that currently, data of this paper are confidential and are not allowed to access (Data and Code Availability Statement.)

### Experiment

All experiments were conducted over 2 (18 subjects) or 3 days (3 subjects.) The experiment consisted of one resting-state session and movement-task sessions. While the resting-state session was performed on each day of the experimental period, we used only the data obtained on the first day, considering the plasticity of the brain activity. The number of movement-task sessions differed between subjects, but at least eight sessions were conducted because of the subjects’ health (e.g., tired and sleepy) and schedule. Seven subjects performed 8 sessions, one subject performed 10 sessions, four subjects performed 12 sessions, one subject performed 15 sessions, and the remainder of the subjects performed 16 sessions (see SI, Table S1 for further details). During experiments, subjects remained in a supine position with their arms fixed to the side of the body. In addition, they wore earphones during the scan period.

### Resting-state session

The resting-state session was conducted before the movement-task session, and 200 images were acquired on each day. Before the resting-state session, subjects were instructed not to think of anything and to focus on the fixation cross on the center of the screen. We used only images that were obtained on the first day (a total of 200 images) to eliminate the after-effects of the movement task. In addition, we denoted the fMRI data in the resting-state session as RS.

### Movement task session

**Fig. 1:**
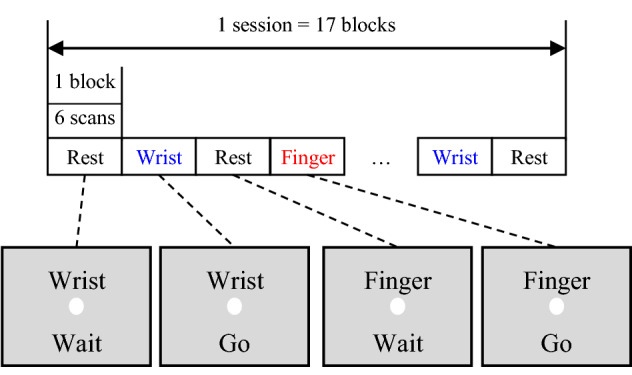
Instruction screen display timing. Note that Wrist, Finger, and Wait were displayed in Japanese in actual

During the movement-task session, we asked subjects to move either their wrist (Wrist task) or finger (Finger task). All movement-task sessions were performed in a block design. Wrist task involved flexion and extension of the right wrist and subjects were instructed not to touch any other body parts (e.g., a thigh) directly prior to beginning the experiment. In Finger task, the fingers of the right hand were flexed and extended. Participants were instructed not to touch the fingers to one another [i.e., subjects did not form a fist, see Figure S1 (b) in SI]. Each movement task was performed in synchrony with a 2-Hz tone delivered via earphones. Each movement task lasted for 18 s, each was performed 4 times per session, with a rest period of 18 s between movement tasks (e.g., Rest period—> Wrist task—> Rest period—> Finger task—> Rest period—> Wrist task…—> Rest period). Thus, the measurement time per session was 306 s (about 5 min) (102 scans). In addition, we presented instructions on a screen as follows: “Wrist” or “Finger” and “Go” or “Wait”, for execution timing (see Fig. 1.) Here, we changed the type of the first movement at the number session (even and odd number session) to eliminate the bias of the movement task. Odd sessions began with the Wrist task, while even sessions began with the Finger task (see SI for task schedule details.) In addition, we denoted the fMRI data in the movement-task session as MT.

### DATA preprocessing

All measured data were preprocessed via MATLAB 2019a (MathWorks, Natick, MA, USA.)

#### Spatial preprocessing

Whole brain images from each session were preprocessed using realignment and head motion corrections (second-degree B-spline), followed by the slice timing corrections using the mean slice of all sessions as a reference via SPM 12 (update revision number: 7487) (Penny, et al. 2011) (http://www.fil.ion.ucl.ac.uk/spm/). T1 images were co-registered to the mean slice via the above-mentioned toolbox and they were segmented to six categories (gray matter, white matter, cerebrospinal fluid, bone, soft tissue, and air.)

#### The region of interest (ROI)

Region of interest (ROI) was defined using the FreeSurfer cortical modeling software package (ver. 6.0.0, Laboratory for Computational Neuroimaging, Athinoula A. Martinos Center for Biomedical Imaging, https://surfer.nmr.mgh.harvard.edu) (Fischl 2012). We defined the ROIs in both contralateral and ipsilateral sensorimotor areas in the cerebral cortex. This is because several studies of human and non-human primate movement have reported that characteristic brain activity during unilateral movement occurs in both the contralateral (Meier et al. 2008) and ipsilateral motor cortices (Tanji et al. 1988; Newton et al. 2005; Verstynen et al. 2005). Furthermore, we also defined the ROIs in the contralateral and ipsilateral cerebellum, because the cerebellum has also been found to be organized somatotopically (Manni and Petrosini 2004). The details of the ROIs defined in the present study are as follows: the primary somatosensory cortex (left and right Brodmann areas 3, 1, and 2): LS1/RS1; the primary motor cortex (left and right Brodmann area 4): LM1/RM1; the pre-motor area (left and right Brodmann area 6): LPM/RPM; and the left and right cerebellar hemispheres: LCB/RCB. Additionally, the ventricles (combined laterals, third, and fourth ventricles) were used as a control ROI. Here, we defined a probabilistic atlas based on cytoarchitecture (Amunts et al. 2007; Fischl et al. 2002; 2007; Hinds 2008; Toga et al. 2006; Zilles et al. 2002) and all ROIs were masked using a gray matter image created by segmentation of SPM12 (Penny et al. 2011), with the exception of the LCB/RCB and the ventricles. The LCB/RCB gray matter area was segmented with FreeSurfer.

#### Temporal preprocessing

After spatial preprocessing of each ROI, components that did not relate to brain signals, such as linear trend or noise components, were eliminated from the time series of each session’s images. These components were eliminated by regressing them out. Here, we defined the noise component (*N*) as that which was derived from signals beyond brain activity (e.g., head motion), consisting of cerebrospinal fluid (CSF), white matter (WM), global signal (GS), and six parameters for realignment of motion correction (RP) [i.e., *N* = (CSF WM GS RP)]. Here, the CSF and WM were defined based on structural images obtained via segmentation implemented in SPM12 and ROI extraction using MarsBar software (ver. 0.44, http://marsbar.sourceforge.net) (Brett et al. 2002) (“Build ROI” in the “ROI definition”; binarization threshold: 0.1 for CSF and WM). Note that the ventricles were eliminated from the CSF signal, because they were used as a control ROI (see below). The GS was defined as that which appeared after BET processing of the structural image obtained via FSL (ver. 5.0.9, the FMRIB Software Library, The University of Oxford, http://www.fmrib.ox.ac.uk/fsl) (Smith et al. 2004) [BET (Smith, Fast robust automated brain extraction 2002) function of FSL was used]. In addition to the noise component *N*, we calculated a temporal derivative of the above noise components of *N* (*N*′) and the quadratic term in each component ($N^{2} N^{{\prime 2}}$). These four components derived from *N* were used as a noise regressor for regressing out ($R_{{{\text{Noise}}}}$) (i.e., $R_{{{\text{Noise}}}} = \left[ {N~N^{\prime}N^{2} N^{{\prime 2}} } \right]$) (Satterthwaite, 2013). The noise regressor was normalized, such that its maximum value was 1. In addition, we defined another regressor that represented the trend and baseline component ($R_{{{\text{Trend}}}}$.) We estimated task components with a regressor derived from a combination of noise and the trend regressor ($R = \left[ {R_{{{\text{Noise}}}} ~R_{{{\text{Trend}}}} } \right]$.) We subtracted this to estimate a task component from the MT.

The RS for each time series were subtracted from that time series’ mean value and a band-pass filter was applied (passband: 0.010–0.10 Hz, fourth order) (Fox et al. 2005). As in the movement task, the noise and trend components of the resting state were estimated and subtracted separately for each day. Note that this noise estimation was conducted independently from data acquisition during the movement task and the resting state session.

### Decoding and cross-decoding

**Fig. 2:**
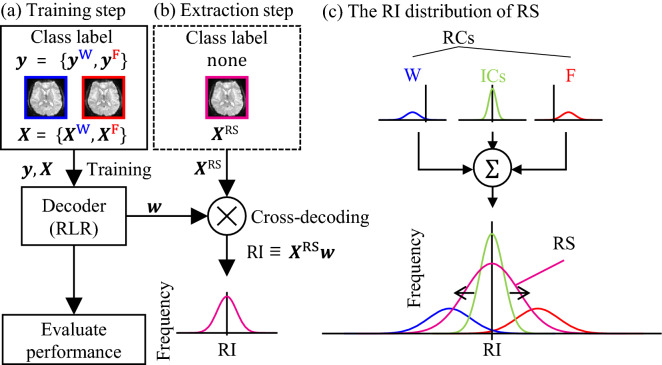
Flowchart of cross-decoding and evaluation. **a**: Training a decoder using regularized logistic regression (RLR) to classify input fMRI data $\user2{X}$ and class label $\user2{y}$ (Wrist task ($\user2{X}^{{\mathbf{W}}} ~{\mathbf{and}}\user2{~y}^{{\mathbf{W}}}$) and Finger task ($\user2{X}^{{\mathbf{F}}} ~{\mathbf{and}}\user2{~y}^{{\mathbf{F}}}$)) (training step). The trained weight parameter $\left( \user2{w} \right)$ was used for the subsequent step. **b** Extracting the task-relevant components (RCs) from resting-state fMRI data $\user2{X}^{{{\mathbf{RS}}}}$ (extraction step). The extraction step outputs a task-relevancy index (RI). **c** Expected RI distribution of the resting state (RS). The resting-state brain activity is constructed by the weighted addition of two distributions: RCs and task-irrelevant components (ICs). In consideration of this assumption, the RI of the resting-state brain activity (magenta line) should be wider than that of the ICs (green line), if the RC exists. The RCs correspond to two distributions that are relevant to each task (Wrist task and Finger task)

To examine the task-similar resting-state brain activity, we used MVPA. In particular, we adopted a variant of MVPA called cross-decoding. In cross-decoding, a decoder is trained to classify brain activity related to each movement task. In general, cross-decoding uses this trained decoder to extract characteristic components from an untrained category (Walther et al. 2011) or different types of one, such as that derived from resting-state imaging (Guidotti et al. 2015). Figure 2 depicts a flowchart of cross-decoding procedures in the present study (see SI for more details on these analyses.)

The cross-decoding is performed in two steps. Below is a summary of the analyses performed during each step.

1. Training step (Fig. 2a)
  Training a decoder using movement data
  Evaluate the trained decoder’s accuracy by leave-one-session-out cross-validation (LOO-CV)
  Check the trained decoder’s weight parameter map in the brain.
2. Extraction step (Cross-decoding; Fig. 2b)
  Extract the RI from the RS using the trained decoder
  Calculate the RI of task-irrelevant component (IC)
  Compare the RI of RS and IC.

Here, RI represents how much the inputted data (RS) are similar to each task's training data (MT). Properties of the RI includes the following:

1. If it is positive on average, the inputted data are similar to the brain activity during the Finger task (i.e., part of RCs).
2. If it is negative on average, the inputted data are similar to the activity during the Wrist task (i.e., part of RCs).
3. If the RI has a peak of distribution at the origin (peak = 0) and is distributed around 0, it is not similar to the brain activity associated with each movement (i.e., IC).

The RS reflects the RC and IC (see Fig. 2c). When the RC is included substantially, the RI of RS (RI_RS_) has a wider distribution, because RI of RC (RI_IC_) is far from the origin (see supplementary for the case that holds this assumption). Moreover, if a brain activity pattern that combined the wrist and finger brain activity input to a trained decoder, the RI does not have wide distribution (see SI for more detail). Therefore, that resting-state fMRI data in the motor-related areas (ROIs) should have the task-similar brain activity if the RI_RS_ in these areas would be wider than motor-unrelated area (i.e., IC.) To test this prediction, we calculated the RI_RS_ and its standard deviation (SD_RS_) as an index of distribution width, and compared the SD_RS_ and SD calculated from task-irrelevant components (see below for details.)

### Training step

#### Training a decoder

First, the first two scans collected for each movement-task trial were excluded to eliminate any effect of the previous task, as hemodynamics are delayed relative to neural signals (Ogawa et al. 1990). Next, data were normalized such that the Euclidean distance between voxels at each time point (scan) was 1. Data for each trial were averaged across the time series. This normalization was used to fit the voxel space during the movement task to resting-state images. Next, the decoder was trained using the normalized data. We used a regularized logistic regression (RLR) approach, which added a regularized L2-norm term. Equations (1)–(3) account for the probability distribution of RLR. Note that $P()$ is the probability function, *y* is a binary variable for the class {Wrist, Finger}, ***X*** is the brain activity pattern (single scan × voxels), and ***w*** is a weighted decoder’s parameter. Furthermore, $\sigma ()$ is the sigmoid function*.* We used the RLR code provided with SLR Toolbox (biclsfy_rlrvar.m) (ver. 1.51, ATR Computational Neuroscience Laboratories, http://www.cns.atr.jp/%7Eoyamashi/SLR_WEB.html) (Yamashita 2011; Miyawaki 2008)

1 $$P\left( {y = {\text{Finger|}}\user2{X},~\user2{w}} \right) = \sigma \left( {\user2{Xw}} \right),$$

2 $$P\left( {y = {\text{Wrist|}}\user2{X},~{\mathbf{w}}} \right) = 1 - \sigma \left( {\user2{Xw}} \right),$$

3 $$\sigma \left( {\user2{Xw}} \right) = \frac{1}{{1 + \exp \left( { - \user2{Xw}} \right)}}.$$

#### Evaluating the trained decoder’s accuracy

Classification accuracy was defined as the percentages of correct class labels (i.e., the number of correctly estimated labels divided by the total number of labels.) Accuracy was calculated using a leaving one session out cross-validation (LOO-CV) for each subject and each ROI. To confirm whether the trained decoder is sufficient to classify each movement, we evaluated its accuracy with a Wilcoxon signed-rank sum test (Wilcoxon 1945) of the difference between each ROI and the Ventricles-ROI at *p* < 0.05. We used a Bonferroni correction to correct for a family-wise error rate of N_ROI_ = 8.

#### Checking the trained decoder’s weight parameter map in the brain

We checked the trained decoder’s weight parameter on the brain to confirm brain activity details and classify each movement task. The values of the weight parameter represent the importance of each voxel in a three-dimensional voxel space. Thus, we generated a map (image) from the weight parameter (weight parameter map). Previous studies have shown that the neural representations related to movement tasks are somatotopically arranged (Penfield and Rasmussen 1952). Thus, we expected that the trained decoder’s weight parameter map might also be organized in a somatotopic manner. However, if weight parameter map revealed a mosaic pattern, we would conclude that brain activity contained complex information.

We transformed all subjects’ weight parameter maps to the MNI 152 coordinates contained in Montreal imaging institute (MNI) space and averaged across all subjects for each ROI. To display voxels that were considered to be particularly important when classifying, we extracted only those voxels with an absolute value of averaged weight parameter map in the top 10% for each movement task.

### Extraction step

#### Extracting the RI from the RS using the trained decoder

Next, we performed cross-decoding to the RS and calculated the RI. Here, RI is defined as the multiplication of the trained decoder’s weight parameter (***w***) and the fMRI data (***X***) (i.e., ${\text{RI}} \equiv \user2{Xw}$). Note that this formula requires the denominator of Pearson correlation coefficient $r_{i} = \frac{{\left( {\user2{x}_{i} - \overline{{x_{i} }} } \right)\left( {\user2{w} - \bar{w}} \right)}}{{SD_{{\user2{x}_{i} }} SD_{\user2{w}} }}$ ($\user2{r}_{i}$: Pearson correlation coefficient at *i*th scan, $\user2{x}_{i}$: fMRI data at *i*th scan, $\overline{{x_{i} }}$: mean value of $\user2{x}_{i}$, $\bar{w}$: mean value of $\user2{w}$, $SD_{{x_{\user2{i}} /\user2{w}}}$: standard deviation of $\user2{x}_{i} /\user2{w}$). The RI was calculated for each scan (time series) and the standard deviation (SD) of the RI was calculated by investigating whether the brain activity during the resting state is similar to that during task performance.

#### Calculating the RI of IC

To evaluate existence of the somatotopic organization for wrists or fingers in the resting-state activity, we considered task-irrelevant components (IC) as null distributions to compare it with RS.

We assumed that an information related to tasks is constructed as multi-voxel patterns, so the RC and IC are different patterns.

However, if there is no limit to the generation of the IC, the IC exists infinitely. Thus, we generated the IC under the constraint of using real fMRI data, and the multi-voxel patterns of data were randomly shuffled to break down the information about each task. Here, the RS was used to generate the IC and the process was replicated 1000 times.

One problem was that the computation cost was too large to generate the IC from each RS scan (number of iterations × number of scans = 1000 × 200), so alternately we randomly shuffled the voxels of RS by each iteration only. This procedure is same shuffling the weight parameter except for the bias term when calculating the RI of IC. Thus, we shuffled the trained decoder’s weight parameter [i.e., the computation cost is the number of iteration (1000)]. Here, the shuffling algorithm we used was the iterative amplitude adjusted Fourier transform (Venema et al. 2006; Venema et al. 2007; Schomburg et al. 2012) (IAAFT). We defined the decoder constructed by this procedure as a shuffled task-relevant decoder (${\text{RD}}^{{{\text{shuffle}}}}$).

#### Comparing the RI of resting-state brain activity and IC

The SD had a bias, which was dependent on the decoder’s accuracy (see SI), and each subject’s decoder was not of the same accuracy in even the same ROI. Thus, we needed to eliminate the bias from the ${\text{SD}}_{{{\text{RS}}}}$ and ${\text{SD}}_{{{\text{IC}}}}$ before comparing each SD. Here, we defined the mean ${\text{SD}}_{{{\text{IC}}}}$ as bias. However, the ${\text{SD}}_{{{\text{RS}}}}$ existed in the ${\text{SD}}_{{{\text{IC}}}}$ distribution, but the mean of the ${\text{SD}}_{{{\text{IC}}}}$ is not the same as SD_RS_ in many cases. Thus, we also subtracted the SD of the ${\text{SD}}_{{{\text{IC}}}}$ distribution to close ${\text{SD}}_{{{\text{RS}}}}$ to the mean of ${\text{SD}}_{{{\text{IC}}}}$. Therefore, we corrected each SD for the following equations:

$${\text{SD}}_{{{\text{RS}}}} = {\text{SD}}_{{{\text{RS}}}} - \left( {{\text{mean}}\left( {{\text{SD}}_{{{\text{IC}}}} } \right) + {\text{SD}}\left( {{\text{SD}}_{{{\text{IC}}}} } \right)} \right),$$

$${\text{SD}}_{{{\text{IC}}}} = {\text{SD}}_{{{\text{IC}}}} - {\text{mean}}\left( {{\text{SD}}_{{{\text{IC}}}} } \right).$$

Finally, we examined whether SD_RS_ is greater than the value at 95% of SD_IC_ distribution.

## Results

### Decoding results

**Fig. 3:**
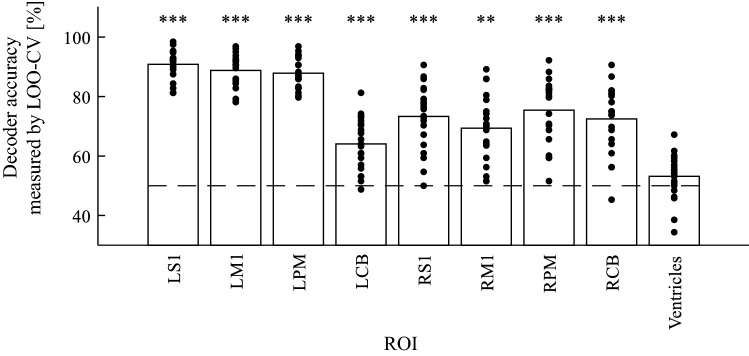
Decoder accuracy measured by leave-one-session-out cross-validation (LOO-CV) across subjects for each ROI. Each dot depicts accuracy for each subject. Horizontal dashed lines depict chance-level accuracy (50%.) The asterisks indicate statistical significance when compared to the Ventricles-ROI; **p < 0.01, ***p < 0.001

We confirmed the trained decoder’s accuracy in each ROI (Fig. 3). The mean accuracy across subjects was as follows: LS1: 90.8 ± 4.9%, RS1: 73.3 ± 10.8%, LM1: 88.8 ± 5.6%, RM1: 69.4 ± 11.1%, LPM: 87.9 ± 5.2%, RPM: 75.4 ± 11.1%, LCB: 64.5 ± 8.8%, RCB: 72.5 ± 11.2%, and Ventricles: 53.2 ± 7.6% (mean ± SD). Results obtained for each motor-related ROI without RM1 were significantly larger than those for the control area (Ventricles-ROI) (*p* < 0.05, corrected). A difference in accuracy between left- and right-lateralized homologous regions (left–right) was observed (S1: 17.5 ± 8.7%, M1: 19.4 ± 11.5%, PM: 12.5 ± 9.7%, CB: − 8.4 ± 9.2%). This result revealed that the accuracy in contralateral ROIs was higher those in ipsilateral ROIs in the cerebrum, while the accuracy in ipsilateral ROI was higher those in the contralateral ROI in the cerebellum, with a difference of approximately over 8%. Additionally, there was no significant difference between subjects with a different number of sessions, except for the RPMdv and one non-anatomical ROI (see below and SI.)

**Fig. 4:**
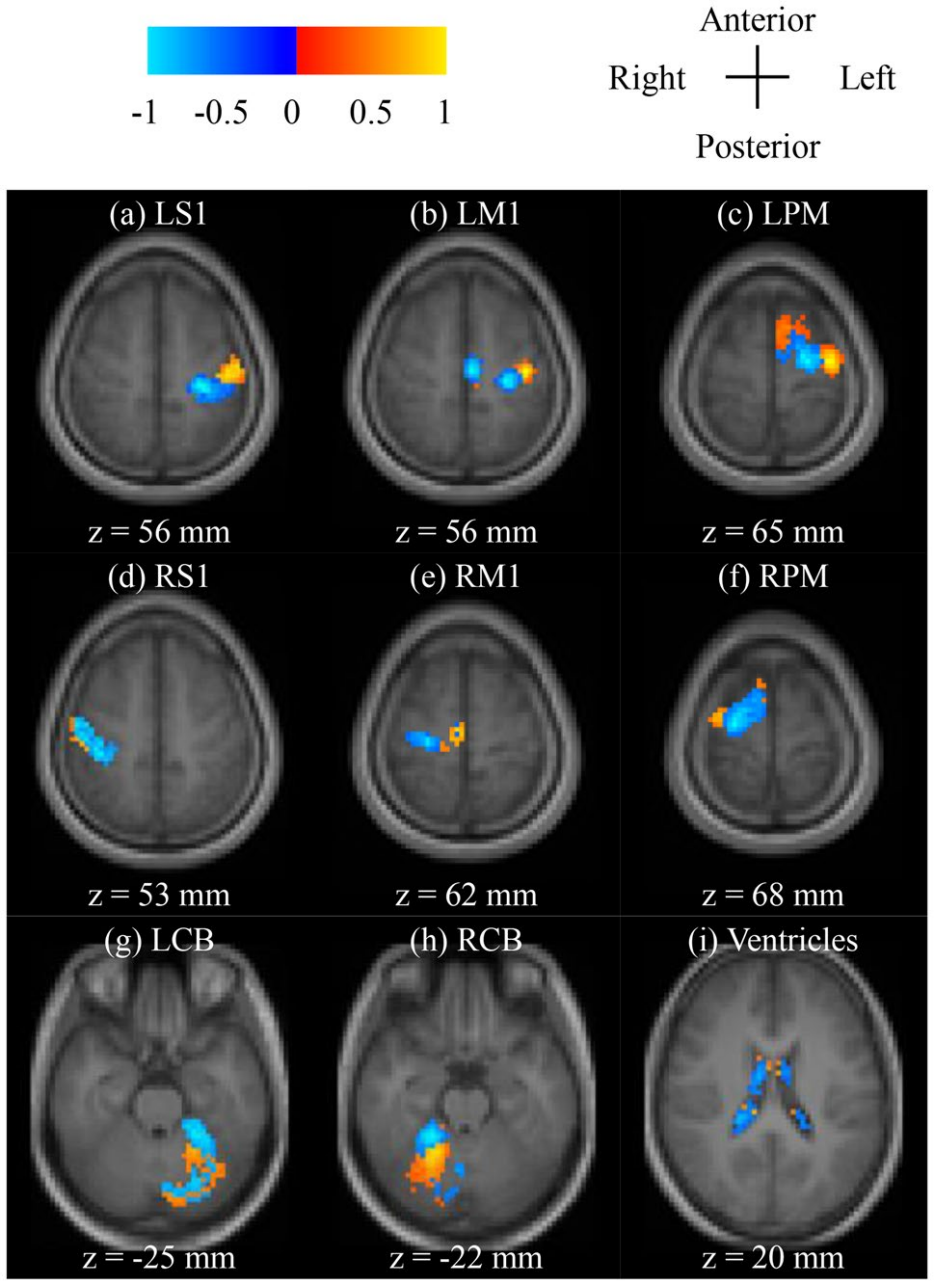
Weight parameter maps for the subject average in each ROI (top 10% of positive/negative values in the weight parameter for each subject) of the trained decoder. Images are visualized on the transverse plane (*Z*). **a**–**h** L/R S1, M1, PM, CB-ROI, (i): Ventricle-ROI. Background structural images (T1-weighted) are normalized to MNI 152 coordinates and averaged across subjects. Warm colors (red to yellow) depict a positive value of voxels, whereas cool colors (sky blue to blue) depict a negative value of voxels

Next, we confirmed the spatial distribution of weights to examine whether the trained decoder’s weight parameter was related to a somatotopic representation. Figure 4 depicts weight parameter maps on a normal brain. Distributions across the left and right hemispheres in cerebrum were similar (Fig. 4a–c, d–f). Consistent with previous studies (Long et al. 2014; Penfield and Rasmussen 1952; Meier et al. 2008; Manni and Petrosini 2004), the wrist and finger activation areas are represented in posterior and lateral regions of the precentral gyrus, respectively. Moreover, the distribution of weight related to each movement was clustered. However, this distribution is observed within a smaller area than previously reported (Wang et al. 2012).

### Cross-decoding results

**Fig. 5:**
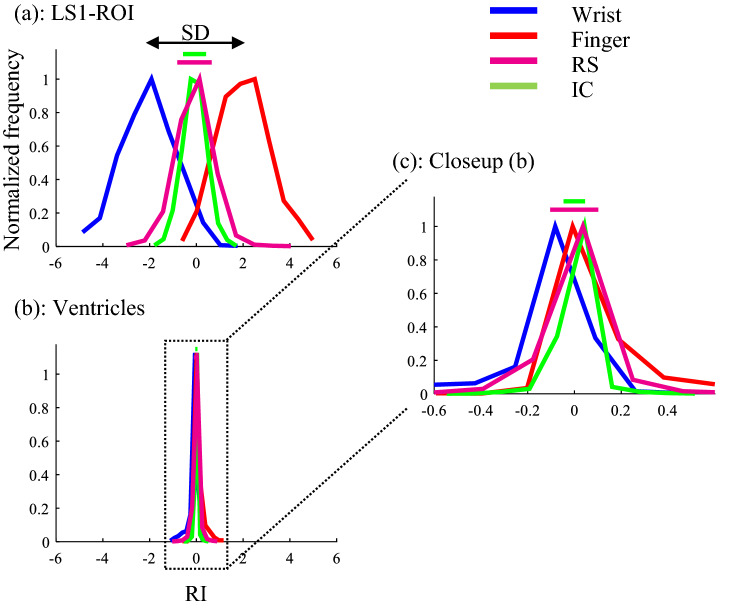
The results of cross-decoding for LS1-ROI (**a**) and Ventricles-ROI (**b**, **c**). **a**, **b** The RI distributions. **c** Enlarged the RI distributions for Ventricles-ROI (**b**). Blue solid line: The RI distribution of Wrist, Red solid line: the RI distribution of Finger, Magenta solid line: the RI distribution of the resting state, and Green solid line: The RI distribution of the task-irrelevant components (top 50th/1000, indicating the value of significance level *p* < 0.05). Upper lines—Magenta: SD of resting state; Green: SD of the task-irrelevant components at top 50th/1000. Note: this SD is before correction

Next, we applied the trained decoder to RS (cross-decoding) and obtained RI distribution for each ROI to confirm RC in the RS. A representative example of the RI distribution for the task in LS1 is depicted in Fig. 5a. The RI_RS_ is widely distributed, with its peak nearly at the origin (= 0), as is expected. Figure 5b depicts the RI distribution of the Ventricles-ROI and Fig. 5c depicts the enlarged that. Unexpectedly, its ${\text{RI}}_{{{\text{RS}}}}$ is widely distributed. We thought that the ${\text{RI}}_{{{\text{RS}}}}$ has many ICs, because Ventricles-ROI is a low accuracy decoder (about chance level.) Therefore, we thought that could not conclude from only RI distribution.

Figure 5a depicts that the RI distributions in LS1 for each task were little overlapping. We found that the overlapping area was wide when the trained accuracy was low and narrow when the accuracy was high.

**Fig. 6:**
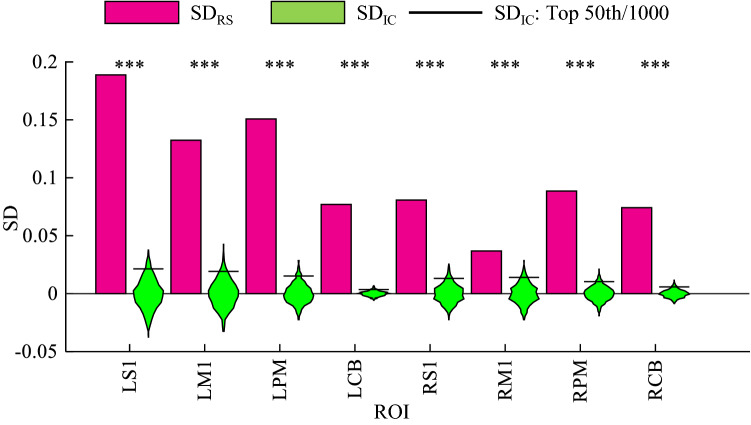
The ${\text{SD}}_{{{\text{RS}}}}$ bar and the ${\text{SD}}_{{{\text{IC}}}}$ violin plot for motor-related regions of interest (ROIs.) The asterisks indicate statistical significance when compared to the ${\text{SD}}_{{{\text{IC}}}}$; ***p < 0.001. Magenta bar: ${\text{SD}}_{{{\text{RS}}}}$; green violin plot: ${\text{SD}}_{{{\text{IC}}}}$; black line: top 50th/1000 of the ${\text{SD}}_{{{\text{IC}}}}$. Note that these ${\text{SD}}_{{{\text{RS}}/{\text{IC}}}}$ are the subtracted mean of ${\text{SD}}_{{{\text{IC}}}}$ and SD of ${\text{SD}}_{{{\text{IC}}}}$ (see the supplementary information for further details.) SD: standard deviation; IC: task-irrelevant component; RS: the resting-state brain activity

Next, we examined SD_RS_ and SD_IC_ for motor-related ROIs and it corrected by Ventricles-ROI. Figure 6 depicts SD_RS_ bar and SD_IC_ violin plot for motor-related ROIs. Their SD_RS_ are: LS1: 0.189 ± 0.212, RS1: 0.081 ± 0.102, LM1: 0.132 ± 0.111, RM1: 0.037 ± 0.076, LPM: 0.151 ± 0.086, RPM: 0.089 ± 0.069, LCB: 0.077 ± 0.042, and RCB: 0.074 ± 0.043 (mean ± SD; arbitrary unit). In addition, the difference in ${\text{SD}}_{{{\text{RS}}}}$ between the left and right hemispheres (left–right) was positive; S1: 0.108 ± 0.186, M1: 0.096 ± 0.124, PM: 0.086 ± 0.131, and CB: 0.003 ± 0.051. This result revealed that, as with accuracy (Fig. 3), the SD of the RI for contralateral ROIs were higher than those of ipsilateral ROIs in the cerebrum. Next, we compared ${\text{SD}}_{{{\text{RS}}}}$ and ${\text{SD}}_{{{\text{IC}}}}$. The results of this test revealed that the ${\text{SD}}_{{{\text{RS}}}}$ for cerebral and cerebellar motor-related activities were significantly larger than the ${\text{SD}}_{{{\text{IC}}}}$; L/R S1, L/R M1, L/R PM, and L/R CB: ~ 0 (*p* value; arbitrary unit). Therefore, we found the task-similar multi-voxel patterns to be bilaterally apparent in cerebral/cerebellar motor-related areas in the resting-state.

## Discussion

In the present study, we examined task-similar multi-voxel patterns in resting-state brain activity compared to movement-induced brain activity. Our results reveal significant similarities between these two conditions and support our hypothesis that neural representations of the resting state in the sensorimotor areas correspond to the fine neural areas activated by movement and have a similar somatotopic arrangement. These results suggest that task-related neural representations in the resting state are finer and more widely distributed in the brain than conventionally expected.

### Somatotopic arrangement in trained decoder’s weight parameter

Several studies have demonstrated that, when executing right-hand movement, the S1, M1, and PM in the contralateral cerebrum (left hemisphere) are activated (Penfield and Rasmussen 1952; Woolsey et al. 1979; Rizzolatti and Luppino 2001) and that these regions are somatotopically organized. Similarly, a somatotopic organization in cerebellar regions has been noted ipsilateral to the movement-executing hand (Lotze 1999; Grodd et al. 2001).

The trained decoder’s weight parameter arrangement was similar (see Fig. 4). In the results presented here, the weight parameter revealed that negative voxels (i.e., wrist-related voxels) were located in the superior–dorsal–medial region and that positive voxels (i.e., finger-related voxels) were located in the superior–dorsal–lateral region of the cerebrum (Fig. 4a–f). These results demonstrate similarities in the somatotopic arrangement of cerebral motor-related areas (L/R S1, L/R M1, and L/R PM-ROI) (Long et al. 2014; Meier et al. 2008; Manni and Petrosini 2004).

Furthermore, we checked the location of the peak value voxel in the weight parameter and the anatomical location using FSL (ver. 5.0.9, the FMRIB Software Library, The University of Oxford, http://www.fmrib.ox.ac.uk/fsl) (Smith et al. 2004) (see Table S6–S8 in SI). The peak value in the motor-related areas was found to be consistent with a somatotopic arrangement for many sensorimotor areas, except for RM1.

The formula of cross-decoding resembles the Pearson correlation coefficient (see SI more detail), and the cross-decoding results show the correlation between RS and the trained decoder’s weight parameter. If the pattern is similar to the somatotopic arrangement, a large RI denotes a strong correlation. From these results, our results are unlikely to be occurred in only somatotopic representation of the weight itself. Also, the resting-state activity that does not have somatotopic representation, but is similar to task-related signals (e.g., the combined activity of the wrist and finger movements) cannot replicate our results (see SI more detail). Therefore, a part of the resting-state brain activity in motor-related areas mimics the somatotopic arrangement.

### Task-related activity in bilateral hemispheres during right-hand movement task

We explored whether a resting-state brain activity pattern is similar to those seen during Wrist and Finger tasks. Our results show that task-similar resting-state brain activity exists. Additionally, from the above relationship between the weight parameter on the brain and the somatotopic representation, we think that a part of resting-state brain activity is similar to the somatotopic arrangement. Moreover, these results were found at both hemispheres in motor-related areas. Several previous studies have established that ipsilateral activation during unilateral movements relates to interhemispheric interactions (Hoshiyama 1997; Kobayashi et al. 2003; Verstynen and Ivry 2011). Similarly, resting-state functional connectivity has also been reported to be bilaterally arranged (Smith et al. 2009). For example, an S1/Cerebellum network is bilaterally detected using independent component analysis (ICA)-based network analysis of the resting state. Consistent with these findings, we also found that task-similar resting-state brain activity was observed in both contralateral and ipsilateral cerebral/cerebellum. In addition, we found that both decoder accuracies and the width of RI distributions (SD_RS_) were greater for ROIs in the dominant hemisphere (contralateral cerebrum ROIs) than in the non-dominant hemisphere (Figs. 3, 6). This suggests that task-similar resting-state brain activity exists similarly (though not identically) and in a more refined degree than has previously been described. In fact, the success of decoding ipsilateral movements have been reported previously using fMRI, electroencephalogram, and electrocorticogram approaches (Fujiwara 2017; Bundy et al. 2012; Scherer et al. 2009; Liu et al. 2010; Hotson 2014; Diedrichsen et al. 2012).

### Difference between task and the resting-state brain activity

As described in the “Introduction”, the previous studies have shown that the resting-state brain activity is similar to that noted during several tasks (Smith et al. 2009; Long et al. 2014; Biswal et al. 1995). Our results with respect to cross-decoding show that the sensorimotor areas (L/R S1, L/R M1, and L/R PM) have significant task-similar brain activity, indicating that our results support those of the previous studies. Contrary to this, other previous studies have shown that resting-state brain activity is not similar to that during task performance and a part of the brain activity during task performance is different from that during the resting state (Arbabshirani et al. 2013; Di et al. 2013; Rehme et al. 2013; Rehme and Grefkes 2013). For example, while performing right-hand movements, the task-related activity in the primary motor cortex in the left hemisphere has a positive correlation with other motor-related areas, whereas that in right hemisphere has a negative correlation with other areas (Rehme et al. 2013). On the other hand, the resting-state brain activity has a positive correlation with bilateral motor-related areas (Rehme et al. 2013 State-dependent differences between functional and effective connectivity of the human cortical motor system). Hence, the correlation of task-state brain activity is asymmetric, but the correlation of that in the resting state is symmetric. Consistent with the previous study (Rehme et al. 2013), in this study, the decoder accuracy in each hemisphere is different; thus, the information for the task-state is asymmetric. However, cross-decoding results in the sensorimotor areas (L/R S1 and L/R PM) in the both hemispheres are approximately the same (*p* value =  ~ 0); thus, there is symmetry. The reason why we obtained symmetric results from the asymmetric decoder is likely the symmetric patterns of the weight parameter map in the decoder. As shown in Fig. 4, the order of positive and negative weight parameter arrangement is symmetric in S1 and PM. Even though the contribution of the positive and negative BOLD responses to the weight is different between LM1 and RM1, the weight parameter map seems to be symmetric (Newton et al. 2005; Mullinger et al. 2014).

In addition, the previous studies (Arbabshirani et al. 2013; Di et al. 2013; Rehme et al. 2013; Rehme and Grefkes 2013) that assessed the difference between task and resting-state brain activity have shown the connectivity between areas (i.e., the analysis target is a large area). On the other hand, our study focused on the intra-regional multi-voxel patterns and cross-decoding is applied to only one ROI (i.e., the analysis target is a small area.) Thus, the scale of the targets of interest (i.e., inter-regional global networks or intra-regional patterns) should be considered to discuss similarity of the task-state and resting-state brain activity. Therefore, we think that the resting-state brain activity is not a simple symmetric pattern, and it may have a pattern that is related to positive and negative BOLD signals as subnetworks in the resting state (Smith et al. 2009).

### Limitations and future directions

Several limitations to the present study warrant some discussion. First, we did not consider overlapping areas that were activated by both wrist and finger-related areas (Meier et al. 2008). Binary MVPA revealed that voxels assigned a positive or negative weight parameter could be used to classify two types of movement. The weight parameter in this area learned as finger (positive value), the wrist (negative value), or neither (nearly zero). Therefore, we have to eliminate this overlapping area to define an ROI (i.e., exclusive OR of wrist and finger-related areas). However, we also have to examine whether this area is like mosaic or arrangement with each movement, because this area might have important information for execution of movements with both body parts.

Additionally, in the present study, we extracted only some of the resting-state data and focused on only two types of movement: that of Wrist and Finger tasks. However, more fine-grained use of specific body parts, such as each finger or each toe, might have instead been used in action tasks. One previous study found that the resting-state functional connectivity is similar to the anatomical connectivity of each finger (Wang et al. 2013). Thus, future work should analyze these more fine-grained representations of the resting-state brain activity using the methods described here.

Our results suggest some potential applications using the resting-state brain activity. Although, in the present study, we only discussed healthy subjects, it might be possible to consider a potential application for patients with other conditions, such as stroke. Generally, performing several movement tasks is hard for the patients. However, there is no active task during the resting-state brain activity measurement. Therefore, analysis of the resting-state brain activity can be used for diagnosis of the state of sensorimotor areas in the patient or tracking changes of these areas during interventions (Lee et al. 2013; Meer 2010; Grefkes 2008). Out results could extend these approaches by improving a resolution of neural representation in the resting state. Needless to say, in its current state, we must confirm whether the trained decoder for different subjects can be used for the analysis of the other subjects. Similarly, there is a possibility that we train a decoder using the current database, and then apply cross-decoding to patients with other conditions.

In the present study, using cross-decoding, the brain activity during the resting state was found to be similar to that during the movement task. However, the brain activity may be task-irrelevant, and thus be common between the movement task and resting state; this would be defined as the common component. From the simulation results, where the brain activity largely contains the common component, only the high accuracy decoder could detect task-relevant information for cross-decoding (see the SI for more information.) We speculated that this could be attributed to the fact that the low accuracy decoder trained using the common component (i.e., without the task-relevant component.) It is best that the common parameter is eliminated before analysis; however, it is difficult to selectively eliminate the common component. Thus, the common component must be considered in future studies.

## Conclusions

In the present study, using a cross-decoding MVPA based on machine learning, we report that task-similar brain activity related to wrist and finger movements exists in the resting state, and these are similar to the somatotopic arrangement for wrist and finger movements. In addition, we confirm that they are specific to motor-related areas of bilateral cerebrum or cerebellum. These results suggest that neural representations in the resting-state may be evaluated in lieu of motor tasks at finer levels in future studies. Therefore, we think that these results will support development of several applications using the resting-state, and will be useful for rehabilitation of patients with other conditions.
